# Draft Genome Sequences of Acinetobacter sp. Strain EKM10A, Enterobacter hormaechei EKM10E, and Enterobacter hormaechei EKM11E (Phylum *Proteobacteria*) Colonizing the Seed Surface Biogel of Echinocystis lobata (Wild Cucumber)

**DOI:** 10.1128/MRA.00184-20

**Published:** 2020-05-14

**Authors:** Eman M. Khalaf, Manish N. Raizada

**Affiliations:** a Department of Plant Agriculture, University of Guelph, Guelph, Ontario, Canada; b Department of Microbiology and Immunology, Faculty of Pharmacy, Damanhour University, Damanhour, Egypt; University of Arizona

## Abstract

Here, we report the draft genome sequences of Acinetobacter sp. strain EKM10A, Enterobacter hormaechei EKM10E, and Enterobacter hormaechei EKM11E, containing 3,978,352, 4,760,222, and 4,758,163 bp, respectively. These seed biogel-associated endophytes were previously isolated from the seed wash of Echinocystis lobata (wild cucumber) and tested *in vitro* for antagonism against soilborne fungal/oomycete pathogens.

## ANNOUNCEMENT

We recently discovered microbes in the well-known biological liquid film (biogel) enclosing seeds of the *Cucurbitaceae* family (e.g., cucumber, pumpkin, cantaloupe). We hypothesize that seed-associated biogels analogously function as the amniotic fluid in humans (1, 2) in protecting the growing embryo against surrounding threats, particularly soil phytopathogens. Seeds were removed aseptically from three fruits (3 replicates) of Echinocystis lobata (wild cucumber), an invasive North American *Cucurbitaceae* species (3), and washed three times using autoclaved double-distilled water (ddH_2_O); then, 100 μl of each wash was streaked onto 3 agar media (LGI [4], potato dextrose agar [PDA], and Reasoner’s 2A agar [R2A]). Three unique bacterial isolates were selected and taxonomically identified using the 16S universal primer pair 799F and 1492R by performing a BLAST search against the NCBI and RDP databases, Acinetobacter sp. strain EKM10A, *Enterobacteriaceae* strain EKM10E, and *Enterobacteriaceae* strain EKM11E (GenBank accession numbers MK852369, MK852366, and MK852384, respectively). Acinetobacter and *Enterobacteriaceae* endophytes are reported to antagonize oomycetes and fungal pathogens (5–7). Therefore, we tested the strains *in vitro* against soilborne pathogens, including two fungi (Fusarium graminearum and Rhizoctonia solani) and two oomycetes (Phytophthora capsici and Pythium aphanidermatum). The strains antagonized the oomycete pathogens *in vitro* (except for Acinetobacter sp. strain EKM10A, which suppressed only *P. capsici*) (8).

Original −80°C glycerol stocks were streaked onto LB agar, and then single colonies were cultured overnight at 37°C and 250 rpm in LB broth, from which genomic DNA was extracted using the DNeasy UltraClean microbial kit (Qiagen catalog number 12224-50), adjusted to 50 ng/μl. TruSeq DNA Nano library prep kits (KAPA HyperPrep kit, number KK8504) were used to construct DNA libraries. EKM10A, EKM10E, and EKM11E generated 2,845,959, 2,699,486, and 3,305,373 raw reads, respectively, in 150-bp paired-end format, using the Illumina NovaSeq 6000 platform. At filtering threshold 30, the resultant clean reads were 2,460,955 (EKM10A), 2,028,274 (EKM10E), and 2,426,387 bp (EKM11E) with maximum scaffold sizes of 622,223, 624,257, and 573,305 bp and minimum scaffold sizes of 207, 204, and 203 bp, respectively. *De novo* assembly was completed using EvoCAT (EVOGENE Clustering and Assembly Toolbox). Using KmerFinder 3.1 (9), reads resulted in 181-, 118-, and 141-fold sequence coverage for EKM10A, EKM10E, and EKM11E, respectively, compared to A. baumannii strain 6200 (GenBank accession number CP010397) and E. hormaechei strain FRM (CP019889) with 85.04%, 82.54, and 82.58% query coverage, respectively. Protein predictions were generated using Prodigal software (10), and predicted proteins were matched against the NCBI nonredundant protein database using BLASTp (Basic Local Alignment Search Tool) software (11). Using InterProScan 5.32-71.0 software, protein domains were calculated (12). Default parameters were used for all software unless otherwise specified. Assembly and annotation statistics are listed in Table 1.

**TABLE 1:** Characteristics and accession numbers of genomes of bacterial endophytes isolated from wild cucumber seed biogel

Isolate	Bacterial species^*a*^	Genome size (bp)	No. of contigs	*N*_50_ (bp)	No. of genes	No. of proteins	G+C content (%)	GenBank accession no.
EKM10A	*A. baumannii*	3,978,352	60	275,334	3,328	3,742	40	JAALLK000000000
EKM10E	*E. hormaechei*	4,760,222	57	505,617	4,236	4,455	56	JAALLL000000000
EKM11E	*E. hormaechei*	4,758,163	47	438,067	4,234	4,442	56	JAALLM000000000

a The taxonomy of bacterial species is according to the updated GenBank databases.

The genome annotations were consistent with their antioomycete activities. All 3 strains possess genes predicted to encode antimicrobial peptides (bacteriocin [13]), phenylalanine and histidine ammonia-lyases (involved in biosynthesis of phenolics and salicylic acid known to trigger antifungal host defense [14]), biosynthesis of acetoin (known to trigger host defense [15]), and chitinase (16). Recent reports have revealed the presence of small amounts of chitin and chitin-like compounds in the cell walls of some oomycetes (17). The *Enterobacter* strains but not the Acinetobacter strain encode cellulase, noteworthy since the oomycete cell wall possesses primarily cellulose (18), along with the *fadJ* gene, which may be involved in degradation of fatty acids, possibly resulting in competitive resource-based inhibition of *Pythium* sp. strains (19).

### Data availability.

The whole-genome shotgun project has been deposited in DDBJ/EMBL/GenBank under the accession numbers noted in Table 1. The raw Illumina reads are available under SRA accession numbers SRR11038236, SRR10961566, and SRR11038209.
